# Investigating the Causal Relationship Between Physical Activity and Chronic Back Pain: A Bidirectional Two-Sample Mendelian Randomization Study

**DOI:** 10.3389/fgene.2021.758639

**Published:** 2021-12-20

**Authors:** Shaowei Gao, Huaqiang Zhou, Siyu Luo, Xiaoying Cai, Fang Ye, Qiulan He, Chanyan Huang, Xiaoyang Zheng, Ying Li, Zhanxin Du, Yaqing Wang, Zhihui Qi, Zhongxing Wang

**Affiliations:** ^1^ Department of Anesthesia, Sun Yat-sen University First Affiliated Hospital, Guangzhou, China; ^2^ Department of Medical Oncology, Sun Yat-sen University Cancer Center, Guangzhou, China

**Keywords:** mendelian randomization, physical activity, chronic back pain, causal inference, instrumental variable

## Abstract

**Background:** Recent observational studies have reported a negative association between physical activity and chronic back pain (CBP), but the causality of the association remains unknown. We introduce bidirectional Mendelian randomization (MR) to assess potential causal inference between physical activity and CBP.

**Materials and Methods:** This two-sample MR used independent genetic variants associated with physical activity and CBP as genetic instruments from large genome-wide association studies (GWASs). The effects of both directions (physical activity to CBP and CBP to physical activity) were examined. Inverse variance-weighted meta-analysis and alternate methods (weighted median and MR-Egger) were used to combine the MR estimates of the genetic instruments. Multiple sensitivity analyses were conducted to examine the robustness of the results.

**Results:** The MR set parallel GWAS cohorts, among which, those involved in the primary analysis were comprised of 337,234 participants for physical activity and 158,025 participants (29,531 cases) for CBP. No evidence of a causal relationship was found in the direction of physical activity to CBP [odds ratio (OR), 0.98; 95% CI, 0.85–1.13; *p* = 0.81]. In contrast, a negative causal relationship in the direction of CBP to physical activity was detected (*β* = −0.07; 95% CI, −0.12 to −0.01; *p* = 0.02), implying a reduction in moderate-vigorous physical activity (approximately 146 MET-minutes/week) for participants with CBP relative to controls.

**Conclusion:** The negative relationship between physical activity and CBP is probably derived from the reduced physical activity of patients experiencing CBP rather than the protective effect of physical activity on CBP.

## Introduction

Back pain, especially low back pain, has become a large burden worldwide, as it is estimated to affect more than 510 million people and cause over 57 million “years lived with disability” in 2016 (Disease, 2018; Wu et al., 2020). At least one-third of patients with back pain report persistent pain after an acute episode and eventually develop chronic back pain (CBP) (Qaseem et al., 2017), which is generally defined as back pain lasting ≥3 months (Deyo et al., 2014). A key step in preventing CBP is the identification of possible risk factors, especially intervenable risk factors. To date, well-known risk factors for CBP have included smoking (Shiri et al., 2010), obesity (Zhang et al., 2018), previous episodes of back pain (Taylor et al., 2014), other chronic conditions (e.g., diabetes, headache) (Ferreira et al., 2013), and poor mental health (Hartvigsen et al., 2018; Power et al., 2001). However, the role of physical activity on CBP is inconclusive (Table 1).

**TABLE 1:** Representative studies for the association between physical activity and chronic back pain.

Study	Type	Design	Region	Time	Sample size	Results	Note	References number
Alzahrani (2019a)	Meta-analysis	Observational studies (cohort or cross-sectional)	Nonspecific	Earliest-March 2017	35 studies, 106,776 participants	Medium physical activity was significantly associated with a lower prevalence of low back pain	This meta-analysis did not specify acute or chronic low back pain	11
Alzahrani (2019b)	Clinical study	Cross-sectional study	Participants form the United Kingdom	1994–2008	60,134 participants	Total PA volume was inversely associated with the prevalence of chronic back conditions	The outcome was chronic back conditions, among which low back pain is one of the most common	13
Shiri (2017)	Meta-analysis	Observational studies (prospective, cohort)	Nonspecific	Earliest-July 2017	36 studies, 158,475 participants	Leisure time physical activity may reduce the risk of chronic low back pain by 11–16%	The exposure was leisure time physical activity	12
Heneweer (2009)	Clinical study	Cross-sectional study	Dutch	1998	3,364 participants	There is some evidence that the relation between physical activity and chronic low back pain is U-shaped	Type of activity (daily routine, leisure time and sport activity), intensity of and time spent on these activities, and back exertion during sport activities were taken into account	18
Kamada (2014)	Clinical study	Cross-sectional study	Japan	2009	4,559 participants	There were no significant linear or quadratic relationships between self-reported physical activity and chronic low back pain	The population were aged 40–79 years	16

Physical activity is defined as musculoskeletal movement that results in energy consumption (Caspersen et al., 1985). As shown in Table 1, recent meta-analyses reviewed tens of observational studies and found a negative relationship between physical activity and CBP (Alzahrani et al., 2019a; Shiri and Falah-Hassani, 2017). A similar conclusion was also reported by other cross-sectional studies (Alzahrani et al., 2019b; B. Amorim et al., 2019). However, studies with high-level evidence (such as randomized control studies), which can address the problem of causal inference, are lacking. Consequently, whether the negative relationship between physical activity and CBP is due to the protective effect of physical activity on CBP or the tendency of patients with CBP to reduce physical activity remains unknown.

Randomized control studies on physical activity are difficult to conduct, as it is unethical to constrain participants’ physical activity. Mendelian randomization (MR) is an alternative method to achieve randomization for this situation by treating genetic variation as a natural experiment in which individuals are randomly assigned to different levels of nongenetic exposure during their lifetime (Davey Smith and Ebrahim, 2003). In addition, MR can strengthen causal inferences by importing a bidirectional design.

**FIGURE 1:**
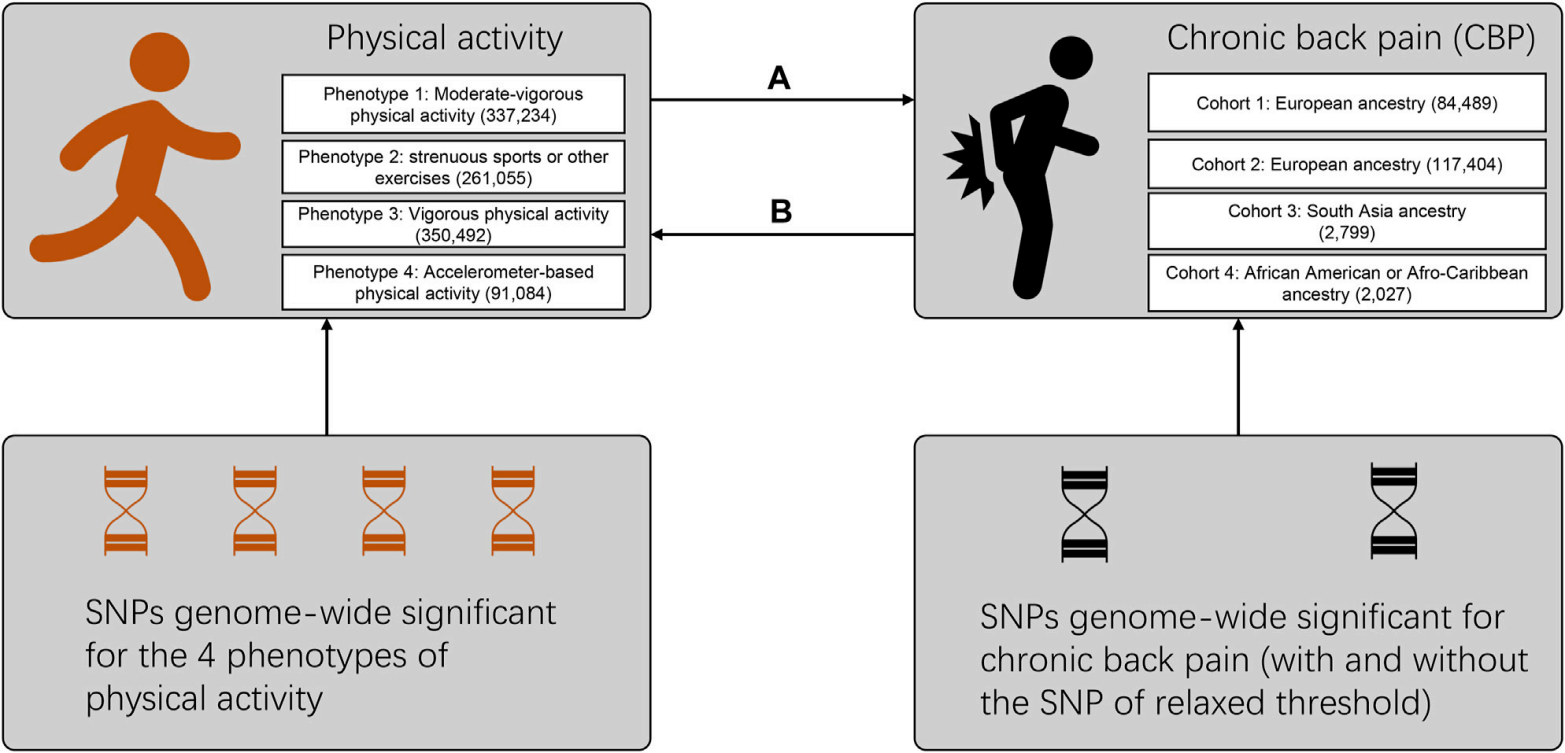
Flow diagram for the design of the bidirectional, two-sample Mendelian randomization study. Multiple phenotypes and cohorts were cross-validated to maintain the robustness of our results. The direction marked **(A)** refers to the effect of physical activity on chronic back pain, while that marked **(B)** refers to the reverse effect. Details on the SNPs used as trait instruments are summarized in Supplementary Tables S2–S4. The numbers of participants for different phenotypes or cohorts are labeled in the brackets. SNP: single-nucleotide polymorphism.

In this study, we first applied bidirectional MR to determine the causal association between physical activity and CBP (Figure 1). We aim to clarify the causal relationship behind this observed negative association between physical activity and CBP. We hypothesize that CBP resulted in reduced physical activity whereas physical activity per se did not have protective effect on CBP.

## Materials and Methods

This is a Mendelian randomization study with a bidirectional and two-sample design, as illustrated in Figure 1. All the data used are summary-level and derived from public genome-wide association studies (GWAS), which had obtained ethical permissions from their respective institutional review boards and written informed consent from their respective participants. Neither patients nor the public were involved in this MR study. The study was conducted under Burgess’s guidelines and reported according to the STROBE-MR statement (Supplementary checklist 2) (Burgess et al., 2019; Davey Smith et al., 2019). We analyzed these data from April 20, 2021 to June 20, 2021.

### Selection of Instruments and Outcome Data

#### Physical Activity

The physical activity instruments were based on Klimentidis’s GWAS conducted with participants of the United Kingdom Biobank cohort (19). This GWAS, using a population of predominantly European ancestry, examined the following four physical activity phenotypes: (Disease, 2018) self-reported moderate-vigorous physical activity [continuous phenotype, 337,234 participants, in standardized units of inverse normalized metabolic equivalent minutes per week (MET-minutes/week)]; (Wu et al., 2020) self-reported vigorous physical activity (binary phenotype, 261,055 participants with 98,060 cases, ≥ 3 vs. 0 for days per week), (Qaseem et al., 2017) self-reported strenuous sports or other exercises (binary phenotype, 350,492 participants with 124,842 cases, ≥ 2–3 vs. 0 for days per week), and (Deyo et al., 2014) seven-day average acceleration from a wrist-worn accelerometer (continuous phenotype, 91,084 participants, in milligravities). The characteristics for each phenotype are summarized in Supplementary Table S1. We chose SNPs from the first phenotype (self-reported moderate-vigorous physical activity) for the primary analysis, as this phenotype yielded the largest number of significant SNPs. To ensure robustness, the SNPs from the other three phenotypes were used in a sensitivity analysis (Supplementary Table S2). In addition, as the GWAS of the accelerometer-based activity identified only two SNPs but had higher heritability than that of the self-reported activity (∼14 vs. ∼5%), the top SNPs meeting a relaxed threshold (*p* < 1 × 10^–7^) were also imported to our study (Supplementary Table S3) in a sensitivity analysis; the method of using SNPs with relaxed thresholds has been used for other MR studies when insufficient SNPs are available (Gage et al., 2017; Hartwig et al., 2017; Choi et al., 2019). We retained only the top independent SNPs by selecting one representative SNP among highly correlated SNPs (r^2^ > 0.001), a process known as “clumping”. If an instrument SNP was not present in the outcome GWAS, then a proxy SNP that was in linkage disequilibrium with the instrument SNPs was searched for instead. Clumping and proxy SNPs are both based on reference data from the 1,000 Genomes Project (Genomes Project et al., 2015).

For the other direction, in which physical activity is regarded as the outcome trait, we again applied Klimentidis’s GWAS (Klimentidis et al., 2018). The completed summary data can be accessed from the OpenGWAS database through the MR-base platform (Elsworth et al., 2020; Hemani et al., 2018a). Similarly, data for all four phenotypes above are available, while moderate-vigorous physical activity was used for the primary analysis.

#### Chronic Back Pain

Genetic instruments for CBP were derived from a genome-wide meta-analysis comprising adults of European ancestry from 16 cohorts (26), in which positive cases were obtained by examining the questionnaires from the participants. These cohorts did not have a consistent definition of CBP: two cohorts used “≥ 1 month of back pain in consecutive years”; nine cohorts used “≥ 6 months of back pain”; six cohorts used “≥ 3 months of back pain”. The control group enrolled participants who reported not having back pain or reported back pain of insufficient duration as cases. Most of the included cohorts did not include question items regarding localization of the pain to the low back or lumbar region specifically. Therefore, a general definition examining chronic “back pain” rather than a more specific chronic “low back pain” definition was applied. This meta-analysis identified four SNPs associated with chronic back pain, one of which met a relaxed threshold (*p* = 3.9 × 10^–7^), while the others met strict criteria (*p* < 5 × 10–8) (Supplementary Table S4). Similarly, we introduced a sensitivity analysis by eliminating the SNP with a relaxed threshold.

For the outcome data, we searched the OpenGWAS database and found four GWAS cohorts with completed summary data (Supplementary Table S5). Two out of the four cohorts are of European ancestry, while the other two contain South Asian populations and African American or Afro-Caribbean populations. Because the MR results may be uninformative for the magnitude (rather than the direction) of the effect when the exposure and outcome studies are derived from different populations (Hemani et al., 2018a), we selected one European ancestry cohort with the maximum sample size (117,404 participants and 80,588 cases) for the primary analysis and the other three for the sensitivity analyses.

### Statistical Analysis

The R package “TwoSampleMR” developed by researchers in the MR-base platform was used for this Mendelian randomization study (Hemani et al., 2018a). Briefly, the algorithm in this package combines the effect sizes of the instruments on exposure traits with those of the instruments on outcome traits using the principle of meta-analysis. In addition to the effect size, the effect allele and its frequency for each instrument—whether for exposure or outcome—must be extracted to determine the direction of the strand.

As the primary method for combining MR estimates, we used the multiplicative random-effect IVW method, which translates to a weight regression of instrument-outcome effects on instrument-exposure effects where the intercept is restricted to zero (Burgess et al., 2013). In this way, bias may occur if horizontal pleiotropy (in which the instruments influence the outcome through causal pathways other than the exposure) is present. We therefore introduced two other MR methods: the weighted median method and MR-Egger regression. The weighted median method chooses the median MR estimate of the instruments as the result, while MR-Egger regression allows the intercept to be a value other than zero (Bowden et al., 2015; Bowden et al., 2016). Both methods are more robust for horizontal pleiotropy, although at the cost of reduced statistical power (Hemani et al., 2018b). Generally, the effect size for the binary outcome should be represented as odds ratio (OR) (i.e., exponentiated β). However, in Klimentidis’s GWAS, a mixed model-model linear regression was used even for binary phenotypes (vigorous PA and strenuous sports or other exercises), leading to unreliable estimates of effect sizes (but not influencing the direction and statistical power) (Klimentidis et al., 2018). We therefore reported the effect estimates in the β value for PA as an outcome trait (we avoided translating the meaning of β for the binary phenotypes) and in the OR for CBP as an outcome trait.

A series of methods were applied for the sensitivity analyses: in addition to setting multiple comparisons among different phenotypes and different cohorts, the funnel plot, Cochran’s Q statistic, leave-one-out analyses, MR-PRESSO, and the MR-Egger intercept test of deviation from the null were used to detect heterogeneity and horizontal pleiotropy (Burgess and Thompson, 2017). By implementing a homonymous R package, MR-PRESSO also detects and corrects outlier SNPs reflecting pleiotropic biases (Verbanck et al., 2018). Finally, to determine potential pleiotropy, we searched each instrument used for the primary analysis in the PhenoScanner GWAS database (version 2; http://phenoscanner.medschl.cam.ac.uk) to find any existing associations with potential confounding traits; then, we removed these SNPs to control the pleiotropic effects and to see if the primary results could be reversed.

## Results

The cohorts used for extracting instruments in the primary analysis were comprised of 337,234 participants for physical activity and 158,025 participants (29,531 cases) for CBP. Details for all parallel cohorts were summarized on the section of Section 2 and Supplementary Tables S1, S5.

### Effect of Physical Activity on CBP

**FIGURE 2:**
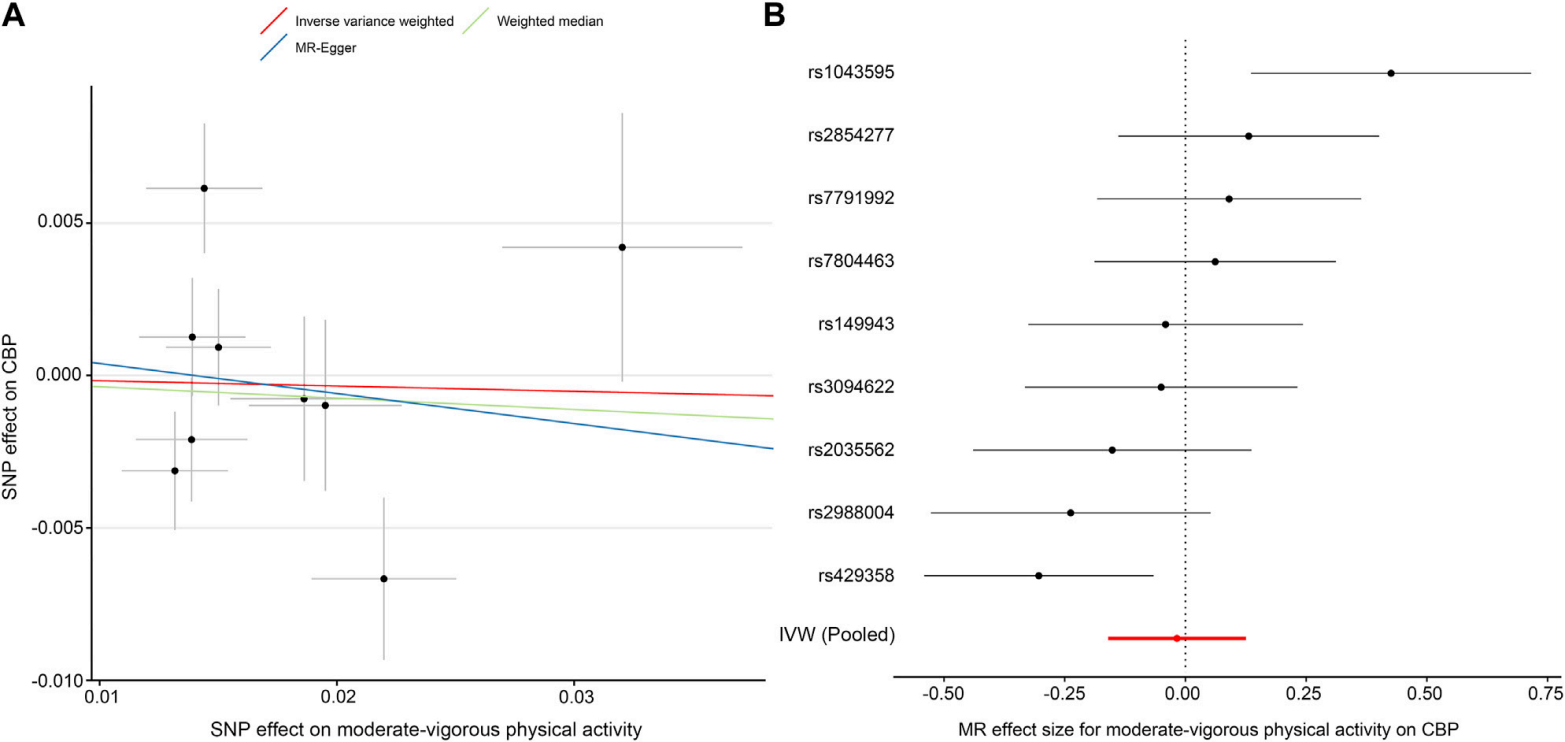
MR plots for the effect of moderate-vigorous physical activity on chronic back pain (CBP). **(A)** Scatter plot of the SNP effect on moderate-vigorous physical activity vs. that on CBP. The slope of each fitted line represents the pooled MR effect calculated by each method. **(B)** Forest plot of individual and pooled MR effect sizes for moderate-vigorous physical activity on CBP. Each point and its corresponding line represent the β value with its 95% CI, respectively. Abbreviations: SNP, single-nucleotide polymorphism; CBP, chronic back pain; MR, Mendelian randomization; IVW, inverse variance weighted.

In this direction, we found no evidence of a discernible causal effect of physical activity on CBP. In our primary analysis—the effect of self-reported moderate-vigorous physical activity on the largest CBP cohort with European ancestry—the combined inverse variance-weighted (IVW) OR was close to 1 (IVW OR, 0.98; 95% CI, 0.85–1.13; *p* = 0.81) (Table 2; Figure 2), which indicated that there is no effect of physical activity on CBP. The results were almost consistent for different exposure phenotypes and different outcome cohorts (Supplementary Table S6). The funnel plot did not detect obvious asymmetry, and the leave-one-out analysis did not change the pattern of the result (Supplementary Figure S1). The MR-Egger intercept test suggested no directional horizontal pleiotropy (intercept, 0.001; standard error, 0.005; *p* = 0.81), even though Cochran’s Q test indicated moderate heterogeneity (*Q* = 19.8; *p* = 0.011). The method of MR-pleiotropy residual sum and outlier (MR-PRESSO) detected one outlier (rs1043595), but the result remained negative when this outlier was removed (Table 2).

**TABLE 2:** MR results for the effect of self-reported moderate-vigorous physical activity on chronic back pain (CBP).

Method	OR (95% CI)^b^	*p* Value	No. of SNPs
With outlier^a^			
IVW	0.98 (0.85–1.13)	0.81	9
Weighted median	0.96 (0.84–1.11)	0.59	9
MR-Egger	0.91 (0.48–1.73)	0.77	9
Without outlier^a^			
IVW	0.94 (0.84–1.05)	0.26	8
Weighted median	0.96 (0.84–1.09)	0.51	8
MR-Egger	1.00 (0.61–1.63)	1.00	8

a The outlier was rs1043595, which was detected with the MR-pleiotropy residual sum and outlier method.

b Indicates odds for CBP per 1-SD increase in moderate-vigorous physical activity (1-SD equals 2084 MET-minutes/week in Klimentidis’s GWAS).

Abbreviations: IVW: inverse variance weighted; CBP, chronic back pain; MR, Mendelian randomization; OR, odds ratio; CI, confidence interval; SNP, single-nucleotide polymorphism.

### Effect of CBP on Physical Activity

**FIGURE 3:**
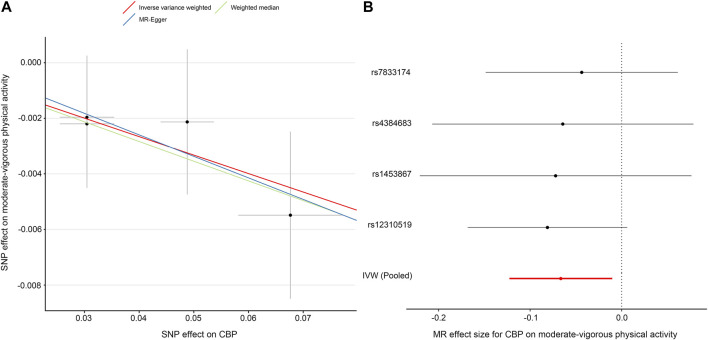
MR plots for the effect of chronic back pain (CBP) on moderate-vigorous physical activity. **(A)** Scatter plot of the SNP effect on CBP vs. that on moderate-vigorous physical activity. The slope of each fitted line represents the pooled MR effect calculated by each method. **(B)** Forest plot of individual and pooled MR effect sizes for CBP on moderate-vigorous physical activity. Each point and its corresponding line represent the β value and its 95% CI, respectively. Abbreviations: SNP, single-nucleotide polymorphism; CBP, chronic back pain; MR, Mendelian randomization; IVW, inverse variance weighted.

In contrast to the previous analysis, we found a robust negative causal relationship between CBP and physical activity. In our primary analysis—the effect of CBP represented by all four single-nucleotide polymorphisms (SNPs) on self-reported moderate-vigorous physical activity—the MR estimate with the IVW method was significantly less than zero (IVW *β*, −0.07; 95% CI, −0.12 to −0.01; *p* = 0.02) (Table 3; Figure 3), implying that participants with CBP tended to reduce their physical activity by approximately 146 MET-minutes/week with respect to those without CBP. The weighted median and MR-Egger tests yielded similar patterns of effects (Table 3). The results were consistent not only with analyses with different outcome traits, such as self-reported strenuous sports and accelerometer-based physical activity, but also with analyses where the SNP with the relaxed threshold was removed for CBP (Supplementary Table S7). The leave-one-out analysis showed that no single SNP was strong for reversely driving the overall effect of CBP on physical activity but detected one SNP (rs12310519) that played a relatively predominant role (Supplementary Figure S2A). Furthermore, the funnel plot presents with a symmetric pattern (Supplementary Figure S2B), and Cohran’s Q test suggested no heterogeneity (Q = 0.3; *p* = 0.96). In addition, MR-PRESSO found no outliers, and the MR-Egger intercept test indicated no consistent pleiotropy (intercept, 0.001; standard error, 0.004; *p* = 0.91).

**TABLE 3:** MR results for the effect of chronic back pain (CBP) on self-reported moderate-vigorous physical activity.

Method	β (95% CI)^a^	*p* Value	No. of SNPs^b^
IVW	−0.07 (−0.12 to −0.01)	0.02	4
Weighted median	−0.07 (−0.13 to −0.01)	0.03	4
MR-Egger	−0.08 (−0.25 to 0.09)	0.47	4

a Indicates a change in multiple of SD of moderate-vigorous physical activity (1-SD equals 2084 MET-minutes/week in Klimentidis’s GWAS) for participants with CBP vs control status.

b No outlier was detected with MR-pleiotropy residual sum and outlier method

Abbreviations: IVW, inverse variance weighted; CBP, chronic back pain; MR, Mendelian randomization; SNP single-nucleotide polymorphism.

### Potential Pleiotropy Searched in PhenoScanner

In total, thirteen SNPs were included in our primary analyses (9 for physical activity to CBP, four for CBP to physical activity). We searched PhenoScanner database for these SNPs and found that the most potential pleiotropy was “trunk fat/fat-free mass”, which was involved in 7/13 of all SNPs (Supplementary Table S8). After removing these involved SNPs, the pattern of the primary results did not change (physical activity to CBP: IVW OR, 1.09; 95% CI 0.85–1.40; *p* = 0.52; CBP to physical activity: IVW β, −0.077; 95% CI, −0.15 to −0.003; *p* = 0.04) (Supplementary Figure S3).

## Discussion

To the best of our knowledge, this is the first MR study to explore the causal relationship between physical activity and CBP. We examined the effects in both directions and found that engaging in more physical activity was not associated with a reduced risk of CBP, but having CBP was associated with reduced physical activity (including both self-reported and accelerometer-based physical activity). The result supports the more intuitive view that the negative association between physical activity and CBP arises from the fact that patients with CBP tend to reduce their physical activity.

### Heritability and Genetics of Selected Variables

The heritability of physical activity varies in terms of different measurements: objective measurement (i.e., accelerometry-based method) has higher heritability than self-reported one (14 vs 5%) (Klimentidis et al., 2018). The study (Klimentidis et al., 2018) we used for extracting instrument SNPs of physical activity applied multi-variable models to adjust covariates such as age, sex, genotyping chip, BMI. This dataset has been involved in several powerful MR studies (Choi et al., 2019; Choi et al., 2020; Papadimitriou et al., 2020), most of which selectively analyzed a few of measurements. To make our results robust, we used all measurements for sensitivity analysis and obtained consistent results, which deeply strengthen our conclusions.

In contrast, the heritability of back pain ranges from 0 to 67%, and is always higher for chronic than acute conditions (Ferreira et al., 2013). The mechanisms of CBP are not only due to anatomic disorders, such as intervertebral disc degeneration, but also to psychological factors. Some previous studies have discovered possible susceptibility genes involved in CBP including SPOCK2, DCC, SLC10A7. (Suri et al., 2018; Freidin et al., 2021). SPOCK2 encodes a protein binding to glycosaminoglycans to form part of the extracellular matrix (Ren et al., 2020), while DCC encodes a transmembrane receptor for netrin-1, an axonal guidance molecule involved in the development of commissural neurons (Finci et al., 2015). SLC10A7, Solute Carrier Family 10 Member 7, is involved in teeth and skeletal development. The evidences above imply that CBP is a complex syndrome, and to some extent related to genetics. The study from which we extracted instrument SNPs of CBP is a meta-analysis including 15 different cohorts, each adjusted for covariates like age, sex, study-specific covariates, and population substructure (Suri et al., 2018). The nature of meta-analysis made the instrument SNPs more robust.

### Comparisons With Previous Traditional Studies

Previous studies reported conflicting results regarding the effect of physical activity on CBP. Some studies showed no association between physical activity and CBP (Kamada et al., 2014; Picavet and Schuit, 2003) or a U-shaped relationship, in which very low and very high levels of physical activity increased the risk of CBP (Heneweer et al., 2009). However, a recent observational study with a large population and two meta-analyses supported a negative relationship between physical activity and CBP (Shiri and Falah-Hassani, 2017; Alzahrani et al., 2019a; Alzahrani et al., 2019b). The observational study involved a population 60,134 adults, but its cross-sectional design was insufficient for identifying the causal inference between physical activity and CBP (Alzahrani et al., 2019b). Although the meta-analysis recruited prospective studies (Shiri and Falah-Hassani, 2017), the observational design was “apt in generating hypotheses and suggesting causality but can never prove it” (De Rango, 2016). In contrast, MR can mimic the design of randomized controlled trials (Hemani et al., 2018a). Given that a SNP is known to be related to a trait (the so-called “instrument variable”), according to Mendel’s law, the alleles at the SNP are causally upstream of the corresponding trait and expected to be random with respect to potential confounders. In an MR study, participants are randomly assigned to the treatment group or control group according to the genotype at the instrument SNP of exposure. Then, the effect size of the causal inference can be calculated as the ratio between the SNP effect on the outcome and the SNP effect on the exposure. Our study extends the current literature from the level of association to the level of causal inference.

### Robustness

Our results were robust to different pairs of exposure and outcome cohorts (Supplementary Tables S6, S7). In the direction of physical activity to CBP, engaging in more physical activity did not significantly change the risk of CBP except in the “ukb-e-3571_AFR” cohort (Supplementary Table S6). The small sample size (approximately 2000 participants) of the “ukb-e-3571_AFR” cohort and the wide range of the OR indicate that the exception probably derives from a random error. In addition, the generalization of our results to different races (e.g., Chinese and African) is limited due to the fact that the exposure and outcome datasets were mostly from European population. Future studies on this issue will require analyses of other races.

In the other direction, from CBP to physical activity, reporting CBP was always associated with reporting reduced physical activity (Supplementary Table S7). However, in the leave-one-out analysis, we found one predominant SNP, rs12310519, without which the OR of reporting CBP on reporting moderate-vigorous physical activity was no longer statistically significant (the 95% CI for the OR included 1) (Supplementary Figure S2A). To examine the extent of the influence of this SNP, we repeated the leave-one-out analysis on the other three phenotypes of physical activity; interestingly, however, this SNP (rs12310519) was not the predominant SNP for self-reported vigorous physical activity and self-reported strenuous sports or other exercises (Supplementary Figure S4). This result may imply different mechanisms by which genetic variance influences different levels of one phenotype.

After looking up the SNPs used for the primary analysis in Phenoscanner database, a potential pleiotropy, “trunk fat/fat-free mass”, was detected (Supplementary Table S8). This trait has been reported as a common predictive factor for both physical activity and CBP (Ness et al., 2007; Urquhart et al., 2011; Brady et al., 2019) and served as an exposure-outcome confounder for the current study. Nevertheless, the pattern of the primary results did not change after controlling this pleiotropy, possibly due to the balance of the multiple SNPs that have effects of different directions on this confounder.

### Limitations

This study has several limitations. First, although different levels of physical activity were included in this study, the CBP was an all-or-none variable. Thus, it was impossible to compare the effect between different levels of CBP. It will be interesting to determine in future studies if the effects of physical activity are similar on different levels of CBP. Second, there were overlapping samples in both the exposure and outcome studies because the physical activity source study and the CBP outcome data both involved participants from the United Kingdom Biobank project. Results from MRs with overlapping samples may be biased due to the winner’s curse phenomenon (Bowden and Dudbridge, 2009). However, we used a sensitivity analysis in which weaker instruments were excluded, which can minimize the bias from sample overlap (Pierce and Burgess, 2013). Finally, the CBP phenotype we used represents a symptom rather than a disease or a biomarker. Compared with other more detailed phenotypes, such as osteoarthritis, additional mechanisms may be involved in CBP, such as muscle injury, nerve root compression, or intervertebral disc degeneration. Thus, a single genome-wide association study is insufficient for finding all SNPs as instruments for CBP. Although the genome-wide meta-analysis we selected for this MR included 16 CBP cohorts, it detected only three to four SNPs, which might partially cover all the mechanisms.

Another point we should clarify is that we used chronic back pain instead of chronic low back pain, a more commonly used phenotype, as the exposure phenotype. The primary reason for this is that the questionnaires used for the included cohorts did not specifically isolate the low back region (Suri et al., 2018)). Given the high agreement between general back pain and low back pain-specific questions (Denard et al., 2010) and since upper/mid back pain without concurrent low back pain is uncommon (Hartvigsen et al., 2009), we believe that our results with CBP can well represent those with chronic low back pain, as exemplified in other studies using similar substitutions (Suri et al., 2018; Suri et al., 2017).

### Importance

Despite these limitations, the MR study performed here provides a novel insight into genetic variants as instruments for assessing the causal inference between physical activity and CBP and obviates typical challenges in observational research while providing an internal explanation for such studies (Koes et al., 2010; Shiri and Falah-Hassani, 2017; Alzahrani et al., 2019a; Alzahrani et al., 2019b). If the negative relationship between physical activity and CBP is truly a reverse causality, the concept that patients with CBP should be engaging in activity, which is recommended by current guidelines (Koes et al., 2010), may need to be reconsidered.

## Conclusion

This study applied MR to examine the causal inference between physical activity and CBP. The negative relationship between these two traits is probably derived from the fact that patients experiencing CBP tend to reduce their physical activities.

## Data Availability

Publicly available datasets were analyzed in this study. This data can be found here: https://gwas.mrcieu.ac.uk/.

## Abbreviations

CBP, chronic back pain; GWAS, genome-wide association studies; IVW, inverse variance-weighted; MR, Mendelian randomization; MR-PRESSO, MR-pleiotropy residual sum and outlier; SNP, single-nucleotide polymorphism.
